# Characterization of Inflammatory Breast Cancer in Hispanic Women from Puerto Rico

**DOI:** 10.7150/jca.77108

**Published:** 2022-10-24

**Authors:** Camiled Quirindongo-Rivera, Valeria Rullán-Varela, Zoe Underill, Mayra Rivera, Karen J. Ortiz-Ortiz, Michelle M. Martínez-Montemayor

**Affiliations:** 1 University of Puerto Rico Comprehensive Cancer Center, San Juan, PR; 2 Public Health Program, Ponce Health Science University, Ponce, PR; 3 Universidad Central del Caribe-School of Medicine, Bayamón, PR; 4 Nicklaus Children's Hospital, Miami, FL; 5 Manati Medical Center, Manatí, PR; 6 Puerto Rico Central Cancer Registry, San Juan, PR

**Keywords:** Inflammatory Breast Cancer, Tumor concordance, Epidemiology, Puerto Rico Central Cancer Registry, Survival

## Abstract

**Background**: Breast cancer (BC) is the most diagnosed cancer and the leading cause of cancer death among women in Puerto Rico (PR). Inflammatory breast cancer (IBC) is considered the most aggressive BC subtype. This study characterized the IBC population of Hispanic women living in Puerto Rico and aimed to estimate the IBC survival rate using data from the Puerto Rico Central Cancer Registry (PRCCR).

**Methods**: This is a retrospective, population-based study using the PRCCR database and the Health Insurance Linkage Database (PRCCR-HILD). We analyzed data from patients that were diagnosed with IBC from January 1, 2008 to December 31, 2018. Patients were identified using the International Classification of Diseases for Oncology, 3rd edition (ICD-O-3) site codes C50.0-C50.9 and histology code 8530. Variables such as age at diagnosis, marital status, health insurance, geographic area of residence, staging variables, tumor receptor subtypes, treatment received, and overall survival (OS) were studied. Statistical analysis methods were employed to describe the population, estimate survival curves and examine the risk of dying.

**Results**: The data of 51 patients were included. The mean age at diagnosis of IBC in the current study was 59 years old. A total of 62.8% of patients had no metastases at diagnosis and 64.7% were diagnosed with stage III disease. Most tumors presented with ER+/PR+/Her2- (21.6%), or a triple negative (ER-/PR-/Her2-, 15.7%) tumor concordance. The OS during the first year was 66% (90% CI: 0.54-0.76), whereas 36 months post-diagnosis was at a low 39% (90% CI: 0.27-0.59). The triple-negative subtype had the worst survival at 36 months (36% [90% CI: 0.11-0.62]). This study revealed through Cox regression analysis that women with stage IV disease and those with ER-/PR- tumor subtype have a higher risk of dying (HR 4.99; [90% CI: 2.30-10.83] and HR 4.74; [90% CI: 1.88-11.95]), respectively.

**Conclusions**: Our results suggest that the Puerto Rican IBC patient population presents unique characteristics. This is the first research to describe the patient profile and characteristics of women diagnosed with IBC in PR. This research increases awareness about this lethal disease in PR.

## Introduction

Inflammatory breast cancer (IBC) is a highly aggressive form of BC 1,2. IBC is a rare and rapidly progressing BC subtype that is classically recognized by the appearance of edema, erythema, and pitting of the skin of the breast. In the United States (US), IBC accounts for 2-5% of all BC but is responsible for 7%-10% of BC mortality 1,2. IBC is reputably threatening, evolving over weeks to months into a severe disease.

IBC has been linked to a younger age at diagnosis 3. The mean age at diagnosis of IBC patients in the US between November 2006 and April 2013 was 51.6 years old, and recently was registered at 57 years old 4. The overall IBC 5-year survival is estimated at 40.5% 5. Despite this grim prognosis, the 5-year survival of patients with IBC has increased over time, probably due to multimodal treatment strategies that include neoadjuvant systemic therapy, followed by radiotherapy and surgery. Nevertheless, IBC's survival rate is still significantly lower than in non-IBC BC patients, which have an overall 5-year survival of 90.3% 6.

Per the American Joint Committee on Cancer (AJCC) TNM system, IBC tumors are designated as T4d at diagnosis 7. The diagnosis of IBC is clinicopathological since it must meet clinical criteria in addition to pathological confirmation of invasive carcinoma. The pathologic assessment of hormone receptors (ER and PR) and HER2/neu tumor receptor status is critical to the staging, the treatment plan, and possibly to the prognosis of the disease. Studies show that triple-negative and ER+/PR+/Her2- IBC tumors have been linked with poorer prognosis 8.

The characterization of IBC in Puerto Rican women has not been studied before. Puerto Rico is a Commonwealth of the United States with an understudied population of more than 3 million Hispanic Americans 9. Thus, the objective of the current study is to describe the epidemiology of the IBC population in Hispanic women in Puerto Rico and estimate their IBC survival rate. Using the Puerto Rico Central Cancer Registry (PRCCR) database complemented with the PRCCR-Health Insurance Linkage Database (PRCCR-HILD), we aim to identify cases of Puerto Rican women diagnosed with IBC.

## Methods

### Data Source

This is a retrospective, population-based study that focuses on the epidemiology and patient profile of IBC in females in PR. This study used data from PRCCR, which is part of the CDC's National Program of Cancer Registries (NPCR) and uses the North American Association of Central Cancer Registries (NAACCR) standards and the Surveillance, Epidemiology, and End Results (SEER) Program for coding data. By law, all health facilities that diagnose or treat cancer patients are required to report each case to the PRCCR 10. The PRCCR collects demographic, clinical, and the first course of treatment information of all cancer cases diagnosed or treated in Puerto Rico and complements this information with the Health Insurance Linkage Database (PRCCR-HILD). PRCCR-HILD includes claims data from the government health plan and the main private health insurance companies for approximately 90% of cancer cases from 2008 onward 10. Recently, the PRCCR received the NAACCR Gold Certification, the highest standard for complete, accurate, and timely data 11. This study was deemed exempt and was approved by the UCC Institutional Review Board # 2018-21.

### Patient inclusion and exclusion criteria

This analysis included women (ages 21-99 years old) diagnosed in PR from January 1, 2008, to December 31, 2018. IBC patients were identified using the International Classification of Diseases for Oncology 3rd edition (ICD-O-3) site codes C50.0-C50.9 and histology code 8530. We only included women that were Hispanic and residents of PR. Therefore, women who were diagnosed or treated in PR but were not PR residents were not included. In addition, cases with an unknown stage at diagnosis were excluded from the survival analysis. Once the IBC patients were identified at the PRCCR, the research team examined and reviewed all cases manually to ensure accuracy in the clinical, staging, and treatment variables. A Certified Tumor Registrar (CTR) from the PRCCR performed quality-control tests and certified the information retrieved from the databases.

### Outcome measures

Demographic variables in the analyses included age at cancer diagnosis (<45, 45-64, and ≥ 65 years), marital status (married, unmarried), health insurance (private, Medicaid, Medicare, and Medicaid/Medicare [dual enrolled]), and geographical area of residence. Geographic regions of Puerto Rico were based on the segregation established by the Puerto Rico Department of Health 12. Clinical variables included: staging variables (pathological tumor size, node, and metastasis), tumor receptor status (ER±, PR±, and Her2±), and tumor receptor subtypes (ER+/PR+/Her2+, ER-/PR-/Her2-, and other combinations). Treatment variables were complemented using the PRCCR-HILD data and included the type of first-course treatment (surgery, chemotherapy, radiotherapy), chemotherapy regimen AC (Doxorubicin with Cyclophosphamide), DD (Dose Dense), FAC (Fluorouracil/Adriamycin/Cyclophosphamide), TCH (Carboplatin/Docetaxel/Trastuzumab), followed by Paclitaxel. We also studied the delay in treatment (<15, 15-44, and ≥45 days), defined as the interval in time from diagnosis to first treatment.

### Statistical Analysis

We used descriptive statistics and frequency analyses to describe the population of IBC patients in Puerto Rico. The Kaplan-Meier method was used to estimate survival curves and the log-rank test to assess differences between survival curves. We used Cox proportional hazards model to examine the effect of demographics and clinical variables on the risk of dying using the hazard ratios (HRs) with 90% confidence intervals (90% CIs). Because our study population is small, p-values can be sensitive 13, thus we decided to use a 0.10 significance level 14. The proportionality assumption was evaluated using Schoenfeld residuals. All analyses were performed using STATA version 17.0 (College Station, TX).

## Results

### General characteristics and pathological description of tumors of IBC patients in Puerto Rico

A total of 51 patients diagnosed with IBC that met inclusion criteria were identified in the PRCCR from January 1, 2008 to December 31, 2018. The mean age at diagnosis was 59 years old. Demographic data for patients with IBC are shown in Table 1. The majority of women were within the age group of 45-64 years old (49%), were unmarried (58.8%), belonged to the Bayamón health region (29.4%), and had Medicaid/Medicaid-Medicare health insurance (51%). A description of all the Puerto Rican municipalities within each health region is depicted in Table 1.

All IBC patients included in this study had tumors with T4d classification following the AJCC cancer staging manual (AJCC, 7ed.). The pathological data in IBC patients are presented in Table 2. Most tumors presented with ER+/PR+/Her2- (21.6%), or a triple-negative (ER-/PR-/Her2-, 15.7%) tumor concordance. Moreover, the most common IBC tumor receptor subtypes were ER+/PR+ (39.2% [n=20]), and ER-/PR- (31.4% [n=16]). In addition, 62.8% of patients displayed no metastases, 54.9% had the involvement of at least one lymph node, and 64.7% were stage III.

### Treatment

A total of 82.4% of IBC patients received treatment (Table 3). Of these, 80.4% received chemotherapy, 62.8% received radiotherapy, and 60.8% had surgery. A total of 49.0% of patients received all three modalities of treatment. The most frequent chemotherapy regimen used was AC (Doxorubicin with Cyclophosphamide), DD (Dose-Dense), FAC (Fluorouracil/Adriamycin/Cyclophosphamide), TCH (Carboplatin/Docetaxel/Trastuzumab), followed by Paclitaxel (data not shown). The average delay in treatment was 15 to 44 days post-diagnosis.

### Survival

**Figure 1:**
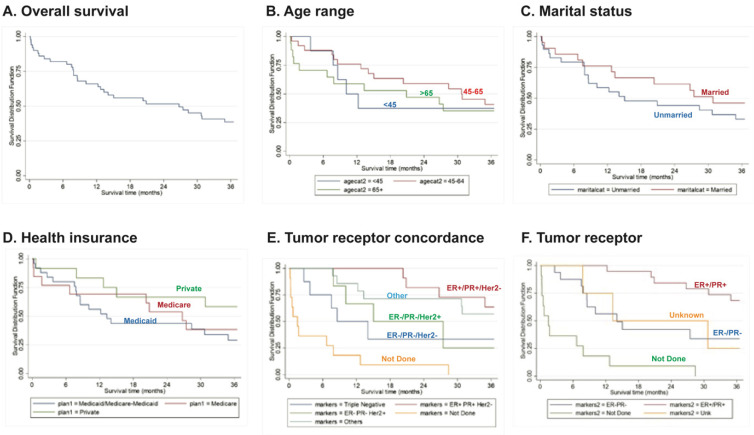
Overall survival curves (A) for patients according to Age (B), Marital Status (C), Health Insurance (D), Tumor Receptor Concordance (E) or Tumor Receptor Status (F). Data derived from the PRCCR from 2008 - 2018. Not done - the patient died before the test was done. Others - includes other tumor concordance combinations. Unknown - not reported.

The OS during the first year post-diagnosis was 66% (90% CI: 0.54-0.76). Importantly, 36 months post-diagnosis, survival drastically reduced to 39% (90% CI: 0.27-0.59) (Fig. 1A). Although not statistically significant, patients within the age range of 45-64 years old had a slightly better survival rate compared to the other age groups during the first year post-diagnosis (76%, 90% CI: 0.58-0.87) (Fig. 1B). At 36 months post-diagnosis, there was increased survival in married women (46% [90% CI: 0.27-0.63]) and in women with private health insurance (58% [90% CI: 0.32-0.77]) (Fig. 1C, D), although these differences were not statistically significant. Importantly, IBC patients with triple-negative (ER-/PR-Her2-) tumors had a significant (*P<*0.0001) overall worse survival rate (36% [90% CI: 0.11-0.62]) (Fig. 1E).

Two Cox proportional hazard regression models were created to study the effects of demographics and different clinical variables for IBC patients. The first model (Table 4) was adjusted for age, tumor stage and tumor receptor subtype. For this analysis, the variables “Not done/unknown” were merged. Our results show that tumor stage IV (HR 4.99; [90% CI: 2.30-10.83]) and ER-/PR- subtypes (HR 4.74; [90% CI: 1.88-11.95]) had a significantly higher risk of death when compared to tumor stage III and ER+/PR+ receptor subtypes, respectively.

Next, we performed a second Cox proportional hazard regression model, which was adjusted for age, tumor stage, health insurance, tumor receptor subtype, and treatment delay (Table 5). Our results show that patients with an age >65 years (HR 0.08; [90% CI: 0.01-0.78]), that had ER-/PR- tumors (HR 6.21; [90% CI: 2.10-18.34]), and had a treatment delay of either <15 days post-diagnosis (HR 6.69; [90% CI: 1.16-38.73]) or ≥45 (HR 4.48; [1.10-18.23] had a higher risk of death**.** Importantly, patients enrolled in Medicare had a significantly increased risk of death (HR 7.54; [90% CI: 1.25-45.59]) when compared to patients with private insurance.

## Discussion

The study presented herein is the first to characterize the patient profile and describe the clinicopathological characteristics of IBC tumors in the Puerto Rican population. Our results suggest that the Puerto Rican IBC patient population presents unique features. Our study shows that for IBC cases diagnosed in PR between 2008 to 2018, the mean age at diagnosis was 59 years old. However, the mean age at diagnosis of IBC for women in the US was 57 years 4. Therefore, when we compare our results with those of women in the mainland US, the women in PR were diagnosed with IBC at a slightly older age during this reported period. Although the women diagnosed in PR were older than in the mainland US, women in PR diagnosed with IBC were younger than those diagnosed with non-IBC BC, whose mean age at diagnosis is 60.5 years 15. Notably, previous population-based studies conducted in the US have found a younger age at onset of IBC among Hispanic women compared to Caucasian women 16-18.

IBC patient tumors that display different molecular subtypes have been linked to distinct outcomes 19. ER+ and Her2+ tumors are associated with better survival when compared to patients with hormone-negative tumors 19. The current study in Puerto Rican women found that 39.2% of IBC patients had ER+/PR+ tumors, while 31.4% of IBC patient tumors were classified as ER-/PR-. A study revealed that IBC patients with ER+/PR+ tumors had a median OS of 31 months when compared to IBC patients with ER-/PR- tumors who had a median OS of 20 months, which is a remarkable difference 20. This is in accordance with our finding that patients with ER-/PR- tumor subtypes displayed a significantly higher risk of death (HR 4.74; [90% CI: 1.88-11.95]) versus IBC patients with ER+/PR+ tumor receptor subtypes. The high incidence of ER-/PR- tumors in the Puerto Rican population is concerning, because the ER-/PR- BC subtype has been linked to poor prognosis and fewer treatment strategies 21,22.

IBC tumors with a triple-negative (ER-/PR-/Her2-) concordance have been associated with the worst outcome 23. In the general population of non-IBC BC in Puerto Rico, triple-negative tumors account for 9.5% of cases 24. Importantly, in our study, an alarming 15.7% of IBC patients presented with triple-negative disease (ER-/PR-/Her2-). In IBC studies, the triple-negative subtype consistently displays the poorest OS 19,20. The higher incidence of triple-negative IBC tumors within the Puerto Rican population results in poorer outcomes measured by a striking worse 3-year OS rate (36% [90% CI: 0.11-0.62]). Similarly, a study in the US found that patients diagnosed between 1996 to 2011 with triple-negative IBC had significantly inferior outcomes compared with non-inflammatory locally advanced triple-negative BC 25. IBC is associated with a poor prognosis due to its high metastatic potential 23 and IBC patients are up to three times more likely to have metastatic disease at the time of diagnosis when compared with patients with non-IBC BC 26. Interestingly, in this study, 62.8% of patients did not have metastatic disease at diagnosis.

Other IBC studies have reported that IBC tumors may lack ER and PR expression but show Her2 amplification 3,23,27. This is not consistent with our study, in which 31.4% of patients were ER-/PR-, but in most cases were Her2- (43.1%), which is another striking finding. A recent study on metastatic IBC reported that Her2+ subtypes displayed the best outcomes without significant differences in hormone receptor status 23. IBC patients with Her2+ tumors have been found to have better OS than those with Her2- tumors, even after adjusting for other prognostic factors 19. Her2+ tumors have targeted therapeutic options, such as trastuzumab. Moreover, the NeOAdjuvant Herceptin (NOAH) trial found that the addition of trastuzumab for women with Her2+ locally advanced BC or IBC resulted in improved event-free survival, OS, and clinical and pathological tumor responses 28.

The treatment for IBC is multimodal, consisting of neoadjuvant chemotherapy, surgery, and radiotherapy. We found that 80.4% of patients received chemotherapy, 62.8% received radiotherapy, and 60.8% underwent surgery. The current consensus regarding surgical management of IBC supports using systemic therapy first then proceeding with surgery once the inflammatory changes have resolved. This strategy is more effective in achieving clear margins upon resection 29-32. We found that the most frequently used chemotherapy agents were AC (Doxorubicin/Cyclophosphamide), DD (Dose-Dense), FAC (Fluorouracil/Adriamycin/Cyclophosphamide), TCH (Carboplatin/Docetaxel/Trastuzumab), followed by Paclitaxel. The current international consensus on the clinical management of IBC supports sequential treatment with AC. In addition, a taxane with or without Carboplatin should be administered before surgery in patients with Her2- IBC 33. For Her2+ IBC, dual anti-HER2-directed therapy with Pertuzumab and Trastuzumab is recommended 33. One recent study in the mainland US found that 80% of patients received trimodal management with chemotherapy, mastectomy, and radiotherapy 32, while in our study, only 49% of patients received all three modalities. This difference should be further studied, to ensure that IBC patients receive the recommended standard of care. Furthermore, the average delay in treatment for patients in our study was 15-44 days. Our study revealed that patients for whom treatment was delayed ≥45 days had a 4.48 risk of death (HR 4.48 [90% CI: 1.10-18.23]). Delay in treatment has been associated with higher mortality 34,35 and timely treatment is critical in the case of a highly aggressive disease such as IBC 36.

The Department of Health in Puerto Rico is segregated in seven health regions: Arecibo, Bayamón, Caguas, Fajardo, Mayagüez, Metro, and Ponce 12. Most IBC cases in this study were found in the health regions of Bayamón and Caguas (29.4% and 23.5% of cases, respectively). No cases were reported in the Fajardo health region, which comprises the Rio Grande, Luquillo, Fajardo, Ceiba, Vieques, and Culebra municipalities. This is believed to be due to the underreporting of cases in this area, as large portions of these areas consist of rural and/or underserved populations. Additionally, the diagnosis of IBC is quite challenging, and, as such, the risk of misdiagnosis is unusually high 2,18,37. For this reason, epidemiological studies such as this one are vital for clinicians to understand a disease process with variable presentation amongst diverse population.

## Conclusions

This is the first study to describe the epidemiology of IBC in the Puerto Rican population. Our study design and the recent study period (2008-2018) are strengths of our study. Furthermore, we obtained the clinical and demographic information from the PRCCR, a center with the highest distinctions ensuring the quality of the data collected. Some limitations of this study include the lack of available information due to the low incidence of IBC in PR, the possible clinical underreporting of cases, and the misclassification of cases due to the heterogeneity of IBC diagnostic criteria and treatment guidelines. Further studies are needed to examine the differences between data we collected and existing data from primarily Caucasian populations. We aim to increase awareness of IBC within our community, encourage physicians in PR to report IBC cases to the PRCCR and disseminate diagnostic guidelines to ensure an improved strategy for early detection of this devastating disease.

## Figures and Tables

**Table 1:** Demographic data of IBC patients

Variables	Number of patients	Percentage (%)
Age at diagnosis		
<45	8	15.7
45-64	25	49.0
>65	18	35.3
Year of diagnosis		
2008-2010	23	45.1
2011-2014	17	33.3
2015-2018	11	21.6
Marital status		
Unmarried	30	58.8
Married	21	41.2
Puerto Rico health region*		
Bayamón	15	29.4
Caguas	12	23.5
Metro	8	15.7
Ponce	8	15.7
Arecibo/Mayagüez*	8	15.7
Health Insurance		
Medicaid/Medicaid-Medicare*	26	51.0
Medicare	13	25.5
Private	12	23.5

*Number of patients that was<6 per category were merged

**Table 2:** Pathological data of IBC tumors

Variables	Number of patients	Percentage (%)
ER		
Positive	19	37.3
Negative	17	33.3
Not done	11	21.6
Unknown	4	7.8
PR		
Positive	17	33.3
Negative	19	37.3
Not done	11	21.6
Unknown	4	7.8
Her2		
Positive	9	17.7
Negative	22	43.1
Not done*	13	25.5
Unknown	7	13.7
Tumor receptor concordance (ER/PR/Her2)		
ER-/PR-/Her2-	8	15.7
ER+/PR+/Her2-	11	21.6
ER-/PR-/Her2+	6	11.8
Not done*	11	21.6
Other**	15	29.4
Tumor receptor subtypes		
ER-/PR-	16	31.4
ER+/PR+	20	39.2
Not done*	11	21.6
Unknown	4	7.8
N		
N0	10	19.6
N1	28	54.9
N2/N3	7	13.7
Nx	6	11.8
M		
M0	32	62.8
M1	15	29.4
Mx	4	7.8
Tumor Stage		
III	33	64.7
IV	16	31.4
Unknown/Other**	2	3.9

*Not done = the patient died before the test could be performed **Other = additional concordance subtype or tumor stage

**Table 3:** Treatment data for IBC cases

Treatment	Number of patients	Percentage (%)
Treatment received		
Yes	42	82.4
No	9	17.7
Chemotherapy		
Yes	41	80.4
No	10	19.6
Radiotherapy		
Yes	32	62.8
No	19	37.3
Surgery		
Yes	31	60.8
No	20	39.2
Chemotherapy first		
Yes	28	54.9
No	23	45.1
Surgery first		
Yes	13	25.5
No	38	74.5
Treatment delay		
<15	7	17.1
15-44	21	51.2
≥45	13	31.7
Chemotherapy first delay		
<15	3	11.1
15-44	16	59.3
≥45	8	29.6

**Table 4:** Multivariate Cox proportional hazards model to examine the effect of demographics and tumor stage and receptor subtypes.

Variable		Hazard ratio	*P*-value	90% Confidence Intervals	
Age	<45	Ref.	Ref.	Ref.	Ref.
	45-64	1.53	0.47	0.58	4.03
	>65	1.48	0.55	0.51	4.28
Tumor stage	III	Ref.	Ref.	Ref.	Ref.
	IV	4.99	**0.00**	2.30	10.83
Tumor receptor subtypes	ER+/PR+	Ref.	Ref.	Ref.	Ref.
	ER-/PR-	4.74	**0.01**	1.88	11.95
	*Not done/ Unknown	15.69	**0.00**	6.09	40.42

Ref. = variables for which the model was adjusted. Variables with significant *p* values are in boldface. *Not done/ Unknown= the patient died before the test could be performed and unknown tumor receptor subtypes

**Table 5:** Multivariate Cox proportional hazards model to examine the effect of demographics, tumor features, health insurance and treatment delay

Variable		Hazard ratio	*P*-value	90% Confidence Intervals	
Age	<45	Ref.	Ref.	Ref.	Ref.
	45-64	0.32	0.26	0.06	1.66
	>65	0.08	**0.07**	0.01	0.78
Tumor stage	III	Ref.	Ref.	Ref.	Ref.
	IV	2.45	0.11	0.98	6.14
Health insurance	Private	Ref.	Ref.	Ref.	Ref.
	Medicaid/Medicare-Medicaid	2.30	0.26	0.69	7.64
	Medicare	7.54	**0.07**	1.25	45.59
Tumor receptor subtypes	ER+/PR+	Ref.	Ref.	Ref.	Ref.
	ER-/PR-	6.21	**0.01**	2.10	18.34
	Not done/ Unknown	7.14	0.19	1.80	28.31
Treatment Delay	15-44	Ref.	Ref.	Ref.	Ref.
	<15	6.69	**0.08**	1.16	38.73
	≥45	4.48	**0.08**	1.10	18.23

Ref. = variables for which the model was adjusted. Variables with significant *p* values are in boldface. *Not done/ Unknown= the patient died before the test could be performed and unknown tumor receptor subtypes.

## Abbreviations

BC: Breast cancer
IBC: Inflammatory breast cancer
PR: Puerto Rico
ER: Estrogen receptor
PR: Progesterone receptor
HER2: Human epidermal growth factor receptor 2
AC: Doxorubicin with Cyclophosphamide
DC: Dose-Dense
FAC: Fluorouracil/Adriamycin/Cyclophosphamide
TCH: Carboplatin/Docetaxel/Trastuzumab
OS: Overall survival
CTR: Certified Tumor Registrar
CSS: cancer-specific survival
BCSS: Breast cancer‐specific survival
HRs: Adjusted hazard ratios
CI: Confidence intervals
TNBC: Triple‐negative breast cancer
PRCCR: Puerto Rico Central Cancer Registry
PRCCR-HILD: PRCCR-Health Insurance Linkage Database

## References

[1] Menta A, Fouad TM, Lucci A, Le-Petross H, Stauder MC, Woodward WA (2018). Inflammatory Breast Cancer: What to Know About This Unique, Aggressive Breast Cancer. Surgical Clinics of North America.

[2] van Golen K (2018). Inflammatory Breast Cancer: A Panoramic Overview. Journal of Rare Diseases Research & Treatment.

[3] Robertson FM, Bondy M, Yang W, Yamauchi H, Wiggins S, Kamrudin S (2010). Inflammatory Breast Cancer: The Disease, the Biology, the Treatment. CA: A Cancer Journal for Clinicians.

[4] Biswas T, Jindal C, Fitzgerald TL, Efird JT (2019). Pathologic complete response (pCR) and survival of women with inflammatory breast cancer (IBC): An analysis based on biologic subtypes and demographic characteristics. International Journal of Environmental Research and Public Health.

[5] Abraham HG, Xia Y, Mukherjee B, Merajver SD (2021). Incidence and survival of inflammatory breast cancer between 1973 and 2015 in the SEER database. Breast Cancer Research and Treatment.

[6] National Cancer Institute (2020). SEER Cancer Stat Facts: Female Breast Cancer. National Institutes of Health.

[7] American Joint Committee on Cancer (2010). AJCC Cancer Staging Manual, Sixth Edition. Vol. 304, JAMA.

[8] Somlo G, Frankel P, Chow W, Leong L, Margolin K, Morgan R (2004). Prognostic indicators and survival in patients with stage IIIB inflammatory breast carcinoma after dose-intense chemotherapy. Journal of Clinical Oncology.

[9] Miller KD, Goding Sauer A, Ortiz AP, Fedewa SA, Pinheiro PS, Tortolero-Luna G (2018). Cancer Statistics for Hispanics/Latinos, 2018. CA: A Cancer Journal for Clinicians.

[10] Torres C, Alvarado M, Ortiz KJ, Zavala D (2020). & Tortolero G. Cáncer en Puerto Rico, 2012-2016.

[12] Departamento de Salud de Puerto Rico Regiones de Salud y Servicios Directos. 2021.

[13] Mitani AA, Haneuse S (2020). Small Data Challenges of Studying Rare Diseases. JAMA Network Open.

[14] Huey T (2010). & Beng T. The correct interpretation of confidence intervals. Proceedings of Singapore Healthcare.

[15] Rodriguez-Velazquez A, Velez R, Lafontaine JC, Colon-Echevarria CB, Lamboy-Caraballo RD, Ramirez I (2018). Prevalence of breast and ovarian cancer subtypes in Hispanic populations from Puerto Rico. BMC Cancer.

[16] Chung-A-Hing A (2022). Differences in Inflammatory Breast Cancer Characteristics and Outcomes Between Caucasian and Hispanic Women in the US [Internet]. 2018 [cited.

[17] Wingo PA, Jamison PM, Young JL, Gargiullo P (2004). Population-based statistics for women diagnosed with inflammatory breast cancer (United States). Cancer Causes and Control.

[18] Hirko KA, Soliman AS, Banerjee M, Ruterbusch J, Harford JB, Merajver SD (2014). A comparison of criteria to identify inflammatory breast cancer cases from medical records and the surveillance, epidemiology and end results data base, 2007-2009. Breast Journal.

[19] Li J, Xia Y, Wu Q, Zhu S, Chen C, Yang W (2017). Outcomes of patients with inflammatory breast cancer by hormone receptor- and HER2-defined molecular subtypes: A population-based study from the SEER program. Oncotarget.

[20] Zhou J, Yan Y, Guo L, Ou H, Hai J, Zhang C (2014). Distinct outcomes in patients with different molecular subtypes of inflammatory breast cancer. Saudi Medical Journal.

[21] Lumachi F (2015). Current medical treatment of estrogen receptor-positive breast cancer. World Journal of Biological Chemistry.

[22] Putti TC, Abd El-Rehim DM, Rakha EA, Paish CE, Lee AHS, Pinder SE (2005). Estrogen receptor-negative breast carcinomas: A review of morphology and immunophenotypical analysis. Modern Pathology.

[23] Dano D, Lardy-Cleaud A, Monneur A, Quenel-Tueux N, Levy C, Mouret-Reynier MA (2021). Metastatic inflammatory breast cancer: survival outcomes and prognostic factors in the national, multicentric, and real-life French cohort (ESME). ESMO Open.

[24] Rosario-Rosado R v, Nazario CM, Hernández-Santiago J, Schelske-Santos M, Mansilla-Rivera I, Ramírez-Marrero FA (2020). Breast cancer in a Caribbean population in transition: Design and implementation of the atabey population-based case-control study of women in the San Juan metropolitan area in Puerto Rico. International Journal of Environmental Research and Public Health.

[25] Biswas T, Efird JT, Prasad S, James SE, Walker PR, Zagar TM (2016). Inflammatory TNBC Breast Cancer: Demography and Clinical Outcome in a Large Cohort of Patients with TNBC. Clinical Breast Cancer.

[26] Fouad TM, Barrera AMG, Reuben JM, Lucci A, Woodward WA, Stauder MC (2017). Inflammatory breast cancer: a proposed conceptual shift in the UICC-AJCC TNM staging system. The Lancet Oncology.

[27] van Laere SJ, van den Eynden GG, van der Auwera I, Vandenberghe M, van Dam P, van Marck EA (2006). Identification of cell-of-origin breast tumor subtypes in inflammatory breast cancer by gene expression profiling. Breast Cancer Research and Treatment.

[28] Gianni L, Eiermann W, Semiglazov V, Lluch A, Tjulandin S, Zambetti M (2014). Neoadjuvant and adjuvant trastuzumab in patients with HER2-positive locally advanced breast cancer (NOAH): Follow-up of a randomised controlled superiority trial with a parallel HER2-negative cohort. The Lancet Oncology.

[29] Yamauchi H, Woodward WA, Valero V, Alvarez RH, Lucci A, Buchholz TA (2012). Inflammatory Breast Cancer: What We Know and What We Need to Learn. The Oncologist.

[30] Curcio LD, Rupp E, Williams WL, Chu DZJ, Clarke K, Odom-Maryon T (1999). Beyond palliative mastectomy in inflammatory breast cancer - A reassessment of margin status. Annals of Surgical Oncology.

[31] Bristol IJ, Woodward WA, Strom EA, Cristofanilli M, Domain D, Singletary SE (2008). Locoregional Treatment Outcomes After Multimodality Management of Inflammatory Breast Cancer. International Journal of Radiation Oncology Biology Physics.

[32] Kell MR, Morrow M (2005). Surgical aspects of inflammatory breast cancer. Breast Disease.

[33] Ueno NT, Espinosa Fernandez JR, Cristofanilli M, Overmoyer B, Rea D, Berdichevski F (2018). International consensus on the clinical management of Inflammatory Breast Cancer from the Morgan Welch Inflammatory Breast Cancer research program 10th anniversary conference. Journal of Cancer.

[34] Cone EB, Marchese M, Paciotti M, Nguyen DD, Nabi J, Cole AP (2020). Assessment of Time-to-Treatment Initiation and Survival in a Cohort of Patients With Common Cancers. JAMA Netw Open.

[35] de Melo Gagliato D, Lei X, Giordano SH, Valero V, Barcenas CH, Hortobagyi GN (2020). Impact of Delayed Neoadjuvant Systemic Chemotherapy on Overall Survival Among Patients with Breast Cancer. The Oncologist.

[36] Khorana AA, Tullio K, Elson P, Pennell NA, Grobmyer SR, Kalady MF (2019). Time to initial cancer treatment in the United States and association with survival over time: An observational study. PLoS ONE.

[37] Dawood S, Merajver SD, Viens P, Vermeulen PB, Swain SM, Buchholz TA (2011). International expert panel on inflammatory breast cancer: Consensus statement for standardized diagnosis and treatment. Annals of Oncology.

